# Solid component ratio influences prognosis of GGO-featured IA stage invasive lung adenocarcinoma

**DOI:** 10.1186/s40644-020-00363-6

**Published:** 2020-12-12

**Authors:** Fenghao Sun, Yiwei Huang, Xiaodong Yang, Cheng Zhan, Junjie Xi, Zongwu Lin, Yu Shi, Wei Jiang, Qun Wang

**Affiliations:** Department of Thoracic Surgery, Zhongshan Hospital, Fudan University, No. 180, Fenglin Road, Shanghai, 200032 China

**Keywords:** Lung adenocarcinoma, Solid component ratio, Ground glass opacity, Prognosis

## Abstract

**Background:**

The computed tomography (CT) characteristic of ground glass opacity (GGO) were shown to be associated with clinical significance in lung adenocarcinoma. We evaluated the prognostic value of the solid component ratio of GGO IA invasive lung adenocarcinoma.

**Methods:**

We retrospectively analyzed the records of GGO IA patients who received surgical resection from April 2012 to December 2015. The solid component ratio was calculated based on thin-slice CT scans. Baseline features were compared stratified by the ratio. Cox proportional hazard models and survival analyses were adopted to explore potential prognostic value regarding overall survival (OS) and disease-free survival (DFS).

**Results:**

Four hundred fifteen patients were included. The higher ratio was significantly associated with larger tumor diameter, pathological subtypes and choice of surgical type. There was a significantly worse DFS with a > 50% ratio. The subgroups of 0% and ≤ 50% ratio showed close survival curves of DFS. Similar trends were observed in OS. Multivariate analyses revealed that the ratio was a significant predictor for DFS, but not for OS. No significant prognostic difference was observed between lobectomy and limited resections.

**Conclusion:**

A higher solid component ratio may help to predict a significantly worse prognosis of GGO IA lung adenocarcinoma.

**Supplementary Information:**

The online version contains supplementary material available at 10.1186/s40644-020-00363-6.

## Synopsis

The higher ratio of solid component was significantly associated with clinicopathological features of GGO patients with IA stage disease, such as tumor diameter and pathological subtype. A higher solid component ratio may help to predict a significantly worse prognosis of GGO IA lung adenocarcinoma.

## Introduction

Lung adenocarcinoma is the most common histological subtype of non-small cell lung cancer with high morbidity and mortality worldwide, which also presents an increasing incidence rate [1]. Early stage lung adenocarcinoma is often detected as showing ground glass opacity (GGO) in thoracic thin-slice computed tomography (CT).

In 2011, the International Association for the Study of Lung Cancer, American Thoracic Society, and European Respiratory Society proposed a new multidisciplinary classification system for lung adenocarcinoma, which were mainly composed of adenocarcinoma in situ, minimally invasive, and invasive adenocarcinoma [2]. The histology subtype and tumor invasion status under the new classification system have significant prognostic impact in lung adenocarcinoma [3–5]. The possible relationships between the radiological features of GGO and clinicopathological features in lung adenocarcinoma have drawn much attention. Our previous work indicated that the CT characteristics of GGO were significantly correlated with histological subtype and gene mutation rate in lung adenocarcinoma [6]. With respect to prognosis, studies also showed that solid components were a negative factor in lung adenocarcinoma [7]. However, Hattori et al. found that solid component size and its corresponding ratio were not prognostic predictors in part-solid clinical N0M0 lung cancer [8]. Therefore, further studies are required to investigate the potential role of the solid components in different subgroups regarding stage and invasion status in GGO-featured lung adenocarcinoma.

In this study, we selected patients with stage IA (The 8th TNM stage of lung cancer) invasive lung adenocarcinoma with GGO imaging features as the study cohort. Patients’ records were reviewed in our institution and analyzed. We explored the clinical and prognostic values of the solid component ratio in early lung invasive adenocarcinoma and provided additional evidence for clinical practice regarding this issue.

## Methods

### Study cohort

This study was approved by the Ethics Committee of Zhongshan Hospital, Fudan University. We retrospectively reviewed records of all GGO patients who received curative surgical resection in our department from April 2012 to December 2015. The selection criteria were described as follows: (1) final pathological report confirmed the diagnosis of invasive lung adenocarcinoma; (2) pathological stages showed IA disease; and (3) R0 resections were achieved. Patients with missing radiological or clinicopathological data were excluded from this study.

Pathological information was acquired from the surgical pathology reports as previously stated [6]. The predominant pattern constituting the largest percentage of pathological findings were defined as the histological subtype. Tumor size was defined as the largest dimension in any plane of the tumor. According to previous reports, the lung adenocarcinoma subgroups were classified into three groups: lepidic predominant type (low - grade), acinar/papillary predominant type (intermediate - grade) and micropapillary/solid predominant type (high - grade) [3, 9–11].

**Fig. 1:**
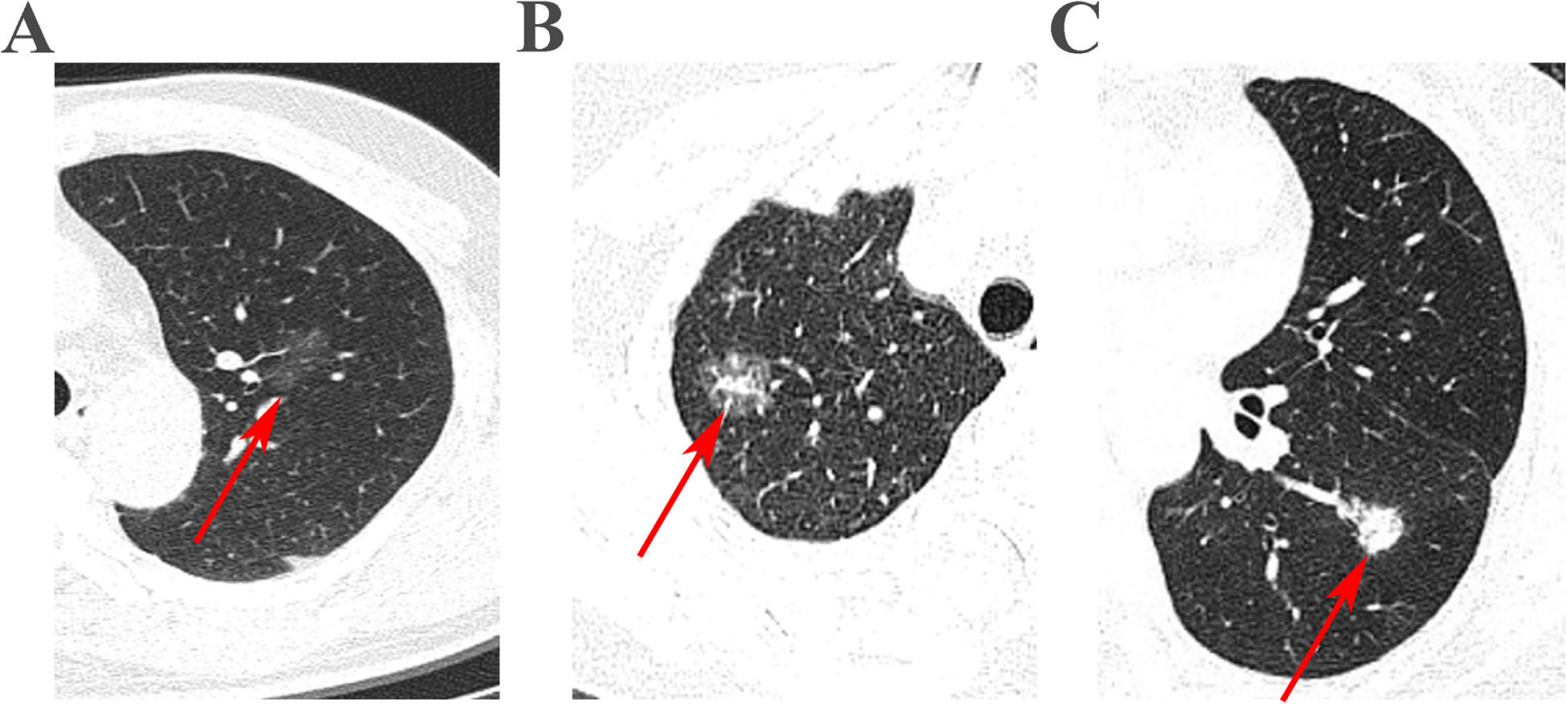
**a** Representative CT scan image of patients with pure GGO. **b** Representative CT scan images of patients with ≤50% solid component. **c** Representative CT scan images of patients with > 50% solid component

CT scans were reviewed by one radiologist and one thoracic surgeon. Disagreements were solved by consensus. The proportion of the solid component was measured from thin-slice CT images (1 mm per slice). CT images were checked on both the mediastinal window (C-40 W400) and lung window (C-600 W1000) settings. The consolidation component was defined as an area of increased opacification, which completely obscured the underlying vascular structures. The solid component ratio was calculated by dividing the maximum consolidation diameter by the maximum tumor dimension including the GGO part in the lung window. To reduce the potential bias in estimating the ratio (percent), we also divided its value into three groups (0%, ≤ 50%, and > 50%). Representative CT scan images of the three classes of cohorts were showed in Fig. 1. To verify the cut-off of the solid component ratio for optimal sensitivity and specificity, we performed ROC analysis and identified that 50% as a cut-off value to group patients has good specificity and sensitivity (Supplementary Figure 1). Based on the solid component ratio, tumors were also classified as pure and mixed GGO subgroups (ratio = 0% or > 0% respectively).

### Statistical analyses

All statistical analyses in this study were performed using R version 3.5.0 (R Foundation for Statistical Computing, Vienna, Austria) and IBM SPSS, version 22.0 (IBM, Inc., Armonk, NY, USA). Baseline characteristics stratified by the solid component ratio were compared using Pearson’s χ2 tests. Overall survival (OS) was defined as the interval from the time of surgery to any death or the last follow-up. Disease-free survival (DFS) was defined as the interval from the date of surgery to the time of the first recurrence or death from any cause or the last follow-up. Survival was calculated by the Kaplan - Meier method, and any survival differences were assessed by using a log-rank test. Univariate and multivariate Cox proportional hazard models were adopted to evaluate the prognostic values of the solid component ratio with respect to both DFS and OS. Differences were considered to be statistically significant if *P* < 0.05.

## Results

A total of 415 patients were included in the study cohort. Baseline clinicopathological information of the study cohort is shown in Table 1. The differences of patients’ clinical factors are also compared and stratified by the subgroup of solid component ratios. As shown in Table 1, the diameters of the resected tumors were significantly larger with the increasing of the solid component ratios (*P* < 0.001). The lung adenocarcinoma subtype was also significantly associated with the solid component ratio (*P* < 0.001).

**Table 1:** Baseline clinicopahtological characteristics of ground glass opacity (GGO) -featured patients with IA stage invasive lung adenocarcinoma stratified by the solid component ratio

Variables	All	Ratio = 0	Ratio ≤ 50%	Ratio > 50%	*P* value
Age	58.34 ± 10.18	56.38 ± 10.69	58.26 ± 10.54	59.76 ± 9.21	0.060
Sex					0.058
Female	260 (62.7)	74 (72.5)	96 (58.9)	90 (60)	
Male	155 (37.3)	28 (27.5)	67 (41.1)	60 (40)	
Tumor diameter	1.58 ± 0.66	1.24 ± 0.54	1.57 ± 0.58	1.81 ± 0.70	< 0.001
Smoking status					0.250
No/unknown	368 (88.7)	95 (93.1)	143 (87.7)	130 (86.7)	
Yes	47 (11.3)	7 (6.9)	20 (12.3)	20 (13.3)	
Surgical type					< 0.001
Lobectomy	323 (77.8)	69 (67.6)	122(74.8)	132 (88)	
Limited	92 (22.2)	33 (32.4)	41 (25.2)	18 (12)	
Histology subtype					< 0.001
Lepidic	64 (15.4)	28 (27.5)	30 (18.4)	6 (4.0)	
Acinar/papillary	344 (82.9)	74 (72.5)	132 (81)	138 (92.0)	
Mircopapillary/solid	7 (1.7)	0 (0.0)	1 (0.6)	6 (4.0)	

**Fig. 2:**
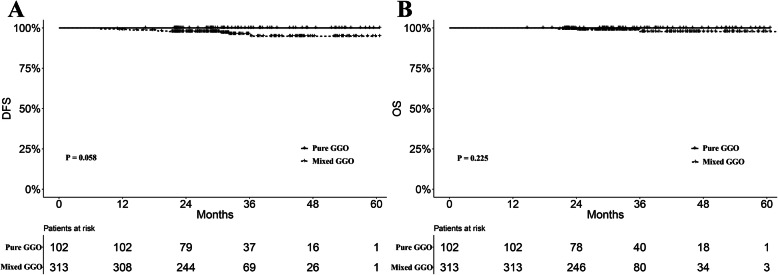
**a** Disease free survival (DFS) curves for all patients stratified by pure and mixed ground glass opacity (GGO) status (*P* = 0.058). **b** Overall survival (OS) curves for all patients stratified by pure and mixed GGO status (*P* = 0.225)

**Fig. 3:**
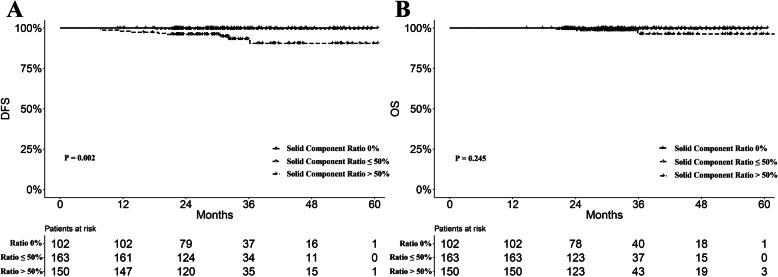
**a** DFS curves for all patients stratified by the solid component ratio (*P* = 0.002). **b** OS curves for all patients stratified by the solid component ratio (*P* = 0.245). Abbreviations are defined in the Fig. 2 legend

The median follow-up time in the entire cohort was 30.8 months. We compared the prognostic difference between pure and mixed GGO subgroups. The mixed GGO subgroup was associated with worse prognosis regarding DFS and OS, although the differences did not achieve statistical significance (*P* = 0.058 and 0.225, Fig. 2a-b). Furthermore, there was a significantly worse DFS in the subgroup with > 50% solid component ratio (*P* = 0.002. Figure 3a). The subgroups with 0% and ≤ 50% solid component ratios showed very close survival curves of DFS (Fig. 3a). Nine patients with > 50% solid component ratios had relapse or metastases, while one patient with metastasis was observed in the ≤50% ratio group. There was no relapse or metastasis found in the pure GGO group. Moreover, similar trends were also observed in the comparison regarding OS (*P* = 0.245, Fig. 3b). Patients with > 50% solid component ratios were associated with worse OS compared with those with ≤50% ratios. There were five deaths in the > 50% ratio group and one death in the ≤50% ratio group. No death was observed in patients in the pure GGO group. Cox regression analyses revealed that the solid component ratio was a significant predictor for DFS (*P* = 0.017, Table 2), but not for OS (*P* = 0.171, Table 3).

**Table 2:** Univariate and multivariate Cox regression analyses of disease free survival for patients with GGO-featured IA stage lung adenocarcinoma. Abbreviations are defined in the Table 1 legend

Variable	HR	95% CI	*P* value	HR	95% CI	*P* value
Age	1.030	0.966–1.099	0.366			
Sex			0.796			
Female	Reference					
Male	1.181	0.333–4.189				
Tumor diameter	0.854	0.352–2.371	0.914			
Smoking status			0.355			
No/unknown	Reference					
Yes	2.077	0.441–9.786				
Surgical type			0.985			
Lobectomy	Reference					
Limited	1.015	0.214–4.807				
Histology subtype			0.894			
Lepidic	Reference					
Acinar/papillary	1.646	0.209–12.996				
Mircopapillary/solid	< 0.001	–				
Solid component ratio			0.017			0.017
0	Reference			Reference		
≤ 50%	> 100	0.001- > 100		> 100	0.001- > 100	
> 50%	> 100	0.001- > 100		> 100	0.001- > 100

**Table 3:** Univariate Cox regression analyses of overall survival for patients with GGO-featured IA stage lung adenocarcinoma. Abbreviations are defined in the Table 1 legend

Variable	HR	95% CI	*P* value
Age	1.146	1.009–1.301	0.036
Sex			0.586
Female	Reference		
Male	1.724	0.243–12.244	
Tumor diameter	0.476	0.090–2.528	0.384
Smoking status			0.383
No/unknown	Reference		
Yes	2.737	0.284–26.333	
Surgical type			0.776
Lobectomy	Reference		
Limited	1.391	0.143–13.542	
Histology subtype			0.888
Lepidic	Reference		
Acinar/papillary	0.570	0.059–5.478	
Mircopapillary/solid	< 0.001	–	
Solid component ratio			0.171
0	Reference		
≤ 50%	> 100	< 0.001- > 100	
> 50%	> 100	< 0.001- > 100	

**Fig. 4:**
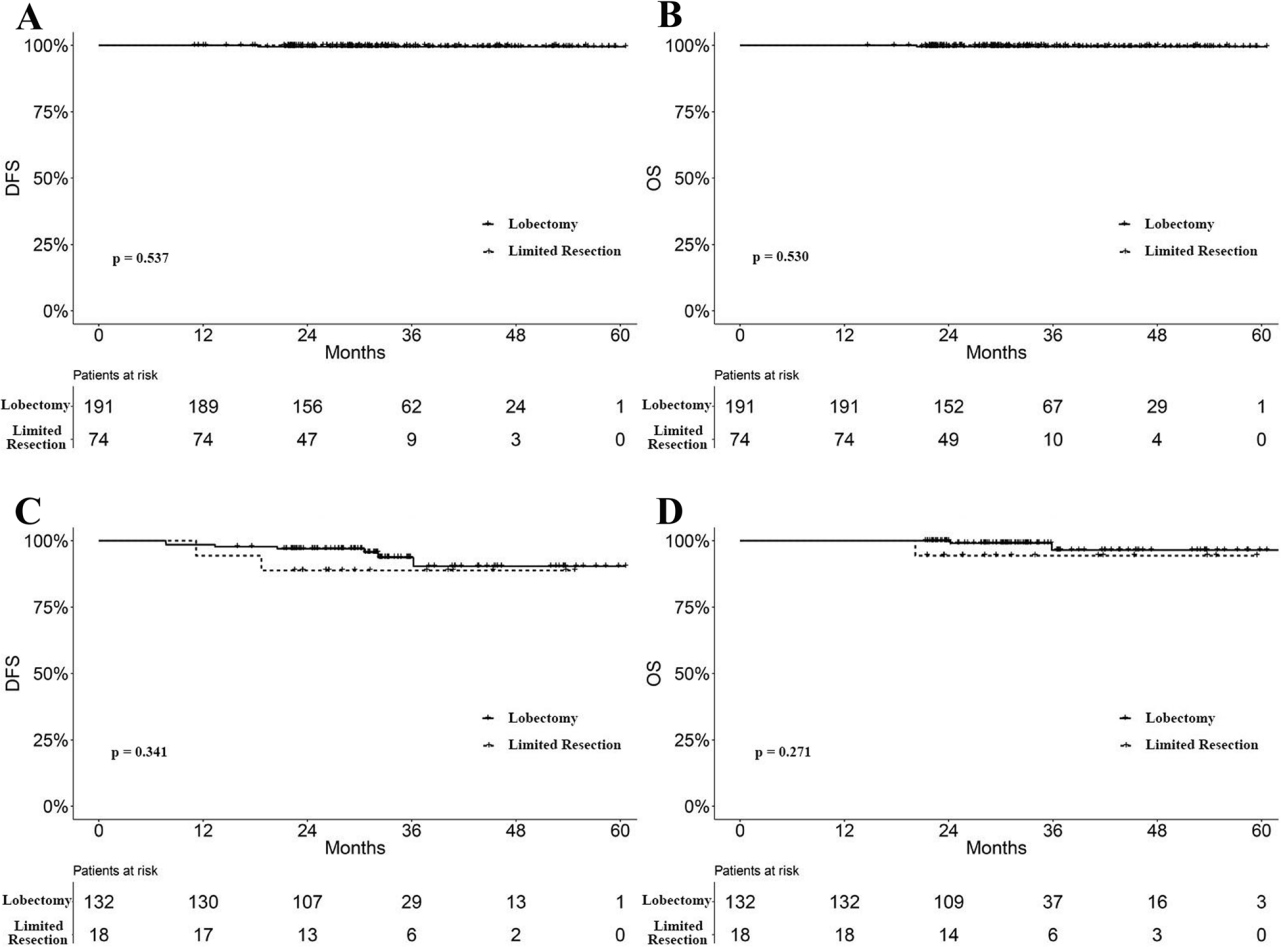
**a** DFS curves of lobectomy and limited resection for patients with 0% or ≤ 50% solid component (*P* = 0.531). **b** OS curves of lobectomy and limited resection for patients with 0% or ≤ 50% solid component (*P* = 0.530). **c** DFS curves of lobectomy and limited resection for patients with > 50% solid component (*P* = 0.341). **d** OS curves of lobectomy and limited resection for patients with > 50% solid component (*P* = 0.271). Abbreviations are defined in the Fig. 2 legend

We also compared the surgical type stratified by the solid component ratio. There was a significantly larger proportion of lobectomy adopted in the > 50% ratio group (*P* < 0.001, Table 1). Survival analyses showed that no significant difference was observed between survival curves of lobectomy and limited resections regarding DFS and OS in patients with 0% or ≤ 50% solid component (*P* = 0.537 and 0.530, Fig. 4a-b). There was also no significantly different prognosis regarding surgical types in patients with > 50% solid components (*P* = 0.341 and 0.271, Fig. 4c-d).

## Discussion

Lung cancer remains a leading cause of cancer deaths all worldwide. Lung adenocarcinoma accounts for the major histological type and GGO is one of its important radiological features. It was reported that the solid component of GGO may represent alveolar collapse and intra-tumor fibrosis [12]. In previous studies, lung adenocarcinoma displayed as GGO features was reported to present a favorable prognosis [13, 14]. Berry et al. found that a small GGO component has significantly better survival than pure solid tumors in resected cN0 lung adenocarcinoma [15]. Part-solid lung adenocarcinoma was also proposed as one special subtype with different clinicopathological features [16]. However, Hattori et al. observed that the solid component ratio and tumor maximum diameter could not predict the OS in radiological part-solid lung cancer [8]. Therefore, the prognostic model of GGO-featured lung adenocarcinoma in different subgroups, such as stage and histology, has not been well established. In our study, we selected GGO-featured patients diagnosed with IA stage invasive lung adenocarcinoma as the study cohort. We reviewed their medical records and evaluated the roles of the solid component ratio. The solid component ratio was significantly associated with clinical factors, like tumor diameter, histological subtype, and choice of surgical resection. Pure GGO tumors showed a trend of better DFS and OS compared with part-solid tumors. Subgroup analyses showed that patients with > 50% solid component ratios had significantly worse DFS. Compared with the group of patients with lower solid components (≤50%), the higher ratio group (> 50%) had a trend of worse DFS, although the difference was not statistically significant.

Huang et al. found that a GGO ratio ≥ 0.75 provided a favorable prognostic prediction in resected lung adenocarcinoma [17]. A cut-off value of 50% solid component ratio was also proposed to predict recurrence of clinical IA stage tumor [18]. In this study, we divided the entire cohort into three subgroups (0%, ≤ 50% and > 50% solid component ratio) to reduce potential bias in estimating the values. We observed that the subgroups of 0% and ≤ 50% solid component ratios shared very similar DFS and OS values. The cut-off value we adopted was only estimated. More advanced calculating tools and statistical algorithm are needed to determine the optimal cut-off value of the solid component ratio.

Lobectomy was recommended as the standard surgical approach for early-stage non-small cell lung cancer [19]. Recently, sublobar resection has been advocated for selected patients [20, 21]. The subgroup are defined by: peripheral nodules sized ≤2 cm with adenocarcinoma in situ and minimally invasive adenocarcinoma, or ≥ 50% ground-glass appearance on CT, or doubling time (≥ 400 days) by radiological surveillance. However, sublobar resection is also a practical choice for patients with advanced age or poor pulmonary function, regardless of the solid component ratio. In GGO-predominant adenocarcinoma Tsutani Y et al. demonstrated that segmentectomy and wedge resection could provide comparable 3-year recurrence-free survival compared with lobectomy [22]. Su H et al. also found that no difference between lobectomy and limited resection was revealed in the GGO-predominant IA stage adenocarcinoma group [18]. Similar results could also be found in previous studies [23, 24]. However, Ye T et al. indicated that wedge resection may be inadequate for invasive lung adenocarcinoma ≤2 cm, with GGO features [14]. In this study, we found that there was no significantly prognostic difference of lobectomy and limited resections stratified by the solid component value of 50%. Further prospective studies with a larger population are needed to explore the optimal surgical choice.

There are also some limitations in our study. First, this was a single-institution retrospective study. Prospective or multi-institutional studies cohorts may be further needed. A larger study cohort may help to identify the prognostic value of solid component ratios in a more detailed subgroup analysis. Moreover, the median follow-up time was 30.8 months in the study cohort. Survival analysis with longer follow-up time could better characterize the prognostic feature of GGO tumors, especially for IA stage disease. However, our study indicated that the solid component ratio was of particular importance in predicting recurrence and survival in the short term after surgery, which may help to improve individualized follow-up plans. Selection bias may also exist due to the exclusion of patients with missing radiological or clinicopathological data in.

## Conclusion

A higher ratio of solid component may help to predict significantly worse prognoses in GGO-featured patients with IA stage lung adenocarcinoma.

## Supplementary Information

**Additional file 1: Supplementary Figure 1.** ROC analysis to determine cut-off of the solid component ratio for optimal sensitivity and specificity.

## Data Availability

The datasets analysed during the current study available from the corresponding author on reasonable request.

## Abbreviations

CT: Computed tomography
GGO: Ground glass opacity
OS: Overall survival
DFS: Disease-free survival
