# Supplementary figures and images for: Solid component ratio influences prognosis of GGO-featured IA stage invasive lung adenocarcinoma
Source: Cancer Imaging. 2020 Dec 12;20:87. doi: 10.1186/s40644-020-00363-6 (PMC7733294; doi:10.1186/s40644-020-00363-6)

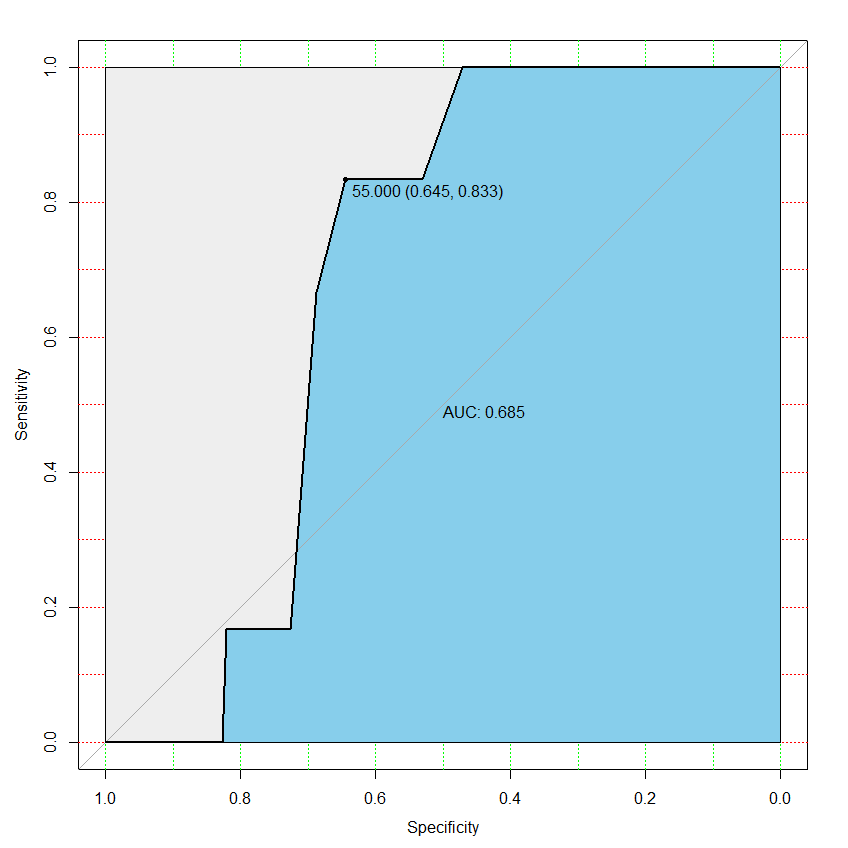

Supplement: Supplementary file 1 — Additional file 1: Supplementary Figure 1. ROC analysis to determine cut-off of the solid component ratio for optimal sensitivity and specificity. [file 40644_2020_363_MOESM1_ESM.png]
